# Influence of Alumina Binder Content on Catalytic Performance of Ni/HZSM-5 for Hydrodeoxygenation of Cyclohexanone

**DOI:** 10.1371/journal.pone.0101744

**Published:** 2014-07-10

**Authors:** Xiangjin Kong, Junhai Liu

**Affiliations:** Shandong Provincial Key Laboratory of Chemical Energy Storage and Novel Cell Technology, School of Chemistry and Chemical Engineering, Liaocheng University, Liaocheng, PR China; Queen’s University Belfast, United Kingdom

## Abstract

The influence of the amount of alumina binders on the catalytic performance of Ni/HZSM-5 for hydrodeoxygenation of cyclohexanone was investigated in a fixed-bed reactor. N_2_ sorption, X-ray diffraction, H_2_-chemisorption and temperature-programmed desorption of ammonia were used to characterize the catalysts. It can be observed that the Ni/HZSM-5 catalyst bound with 30 wt.% alumina binder exhibited the best catalytic performance. The high catalytic performance may be due to relatively good Ni metal dispersion, moderate mesoporosity, and proper acidity of the catalyst.

## Introduction

The deoxygenation reduction of carbonyl compounds is among the most important and prevalent reactions in the pharmaceutical industry, fine chemical industry, as well as in the production of bio-oils from biomass. Recently, hydrodeoxygenation has drawn people’s attentions as a green approach for this transformation [1]–[3]. In early studies, metal supported on solid acid, especially on HZSM-5 has proved to be an efficient catalytic system for this method [4]. The catalyst supported on HZSM-5 is often prepared in powder form, and needs to be shaped into bodies such as spheres, granules and extrudates (Ex) prior to their usage in commercial reactors for the sake of avoiding pressure drop and achieving high mechanical strength. In general, the shaped catalyst was prepared with the addition of binder, and alumina is among the most commonly employed one [5]–[7]. However, the amounts of alumina binder may play an important role in the physicochemical properties of the catalyst, as well as the catalytic performance Thus, particular attention should be paid to the influence of alumina binder content on catalytic properties and performance for use of such a catalyst at the industrial level. However, to the best of our knowledge, there has been few reports on this.

In this contribution, Ni/HZSM-5 catalysts with different amounts of alumina binder were prepared and characterized by N_2_ sorption, X-ray diffraction, H_2_-chemisorption and temperature-programmed desorption of ammonia. And, the obtained results are presented in the following section.

## Experiments

### Materials and catalysts

The parent HZSM-5 powder (nominal Si/Al = 25) was provided by Nankai University catalyst Co., Ltd. All the other chemicals in the present studies were reagent grade and were used without further purification.

The supported monometallic catalysts with different amounts of alumina binder were prepared by a two step method. First, the Ni species precursor was obtained by coprecipitation method as described in our previous work [8]. And then obtained precursor was kneaded with HZSM-5 as well as the alumina binder. For example, Ni_20_/HZSM-5-30% (the subscript 20 means the content of Ni in the catalyst was 20 wt%; 30% means the content of alumina binder in the support was 30 wt%.) was prepared as follows: 16.18 g metal species precursor were kneaded with a mixture of 22.40 g HZSM-5 and 14.44 g pseudo-boehmite (alumina source), accompanied with deionized water as adhesive, and then molded to bars (diameter: about 3 mm) with an extruder. After being dried in air for 6 h at 110°C, the bars were calcined for 4 h at 500°C.

Catalysts used during this investigation are denoted as: ZA10, ZA30, ZA50 and ZA100, where Z, A and the number represent Ni_20_/HZSM-5, alumina binder and binder content (wt.% in support), respectively. A non-agglomerated Ni_20_/HZSM-5 catalyst as reference was also prepared and named as ZA0.

### Catalysts characterization

Brunner-Emmett-Teller (BET) surface area and pore volume were measured by N_2_ sorption at 77 K with a NOVA 2000 e surface and porosity analyzer (Quantachrome, US). The loading of Ni on the catalysts were identified by inductively coupled plasma analysis (ICP) on a Varian 710-ES spectrometer. H**_2_**-chemisorption was carried out on a TP-5000 instrument from Xianquan Ltd. The acid capacity was investigated via temperature-programmed desorption of ammonia (NH_3_-TPD) using a TP-5000 instrument at the atmospheric pressure with a thermal conductivity detector (TCD) device. Powder X-ray diffractions (XRD) were recorded on a Rigaka D/max 2500 X-ray diffractometer with Cu-K_α_ radiation (40 kV, 100 mA) in the range of 5–90°.

### Catalytic reaction

The reaction was carried out in a fixed-bed tubular reactor with an inner diameter of 15.0 mm and a length of 550.0 mm, which was loaded with 18.0 g catalyst. All the catalysts were reduced at 360°C for 4 h in a stream of hydrogen under 1.0 MPa before use. A solution of cyclohexanone (concentration in 1,4-dioxane: 20.0 wt %.) was dosed into the reactor by a syringe pump. The continuous reaction was conducted at 160°C under H_2_ pressure of 2 MPa, GHSV 150 h^−1^ (pure H_2_), LHSV 0.6 h^−1^. The reaction mixture was collected using a cold trap (0–2°C) and analyzed without preliminary separation by off-line gas chromatograph (OV-1701 capillary column: 30 m×0.25 mm, 0.2 µm film thickness). The components of the reaction mixture were identified by GC/MS (HP-1 capillary column: 30 m×0.25 mm, 0.2 µm film thickness) which was equipped with an ion-trap detector.

## Results and Discussion

### Textural properties of the catalysts

**Figure 1:**
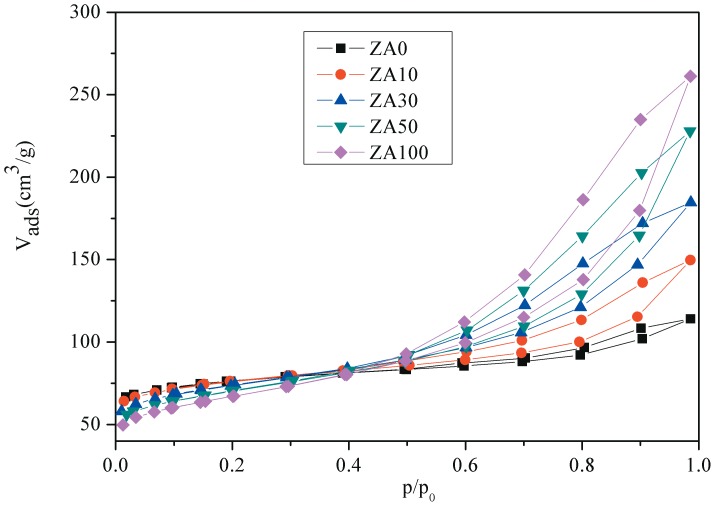
Nitrogen adsorption-desorption isotherms of different samples. The uptake of N_2_ of these catalysts was gradually increased with the increases in binder content which contains a large number of mesopores created by inter-crystalline voids of alumina crystallites.

**Figure 2:**
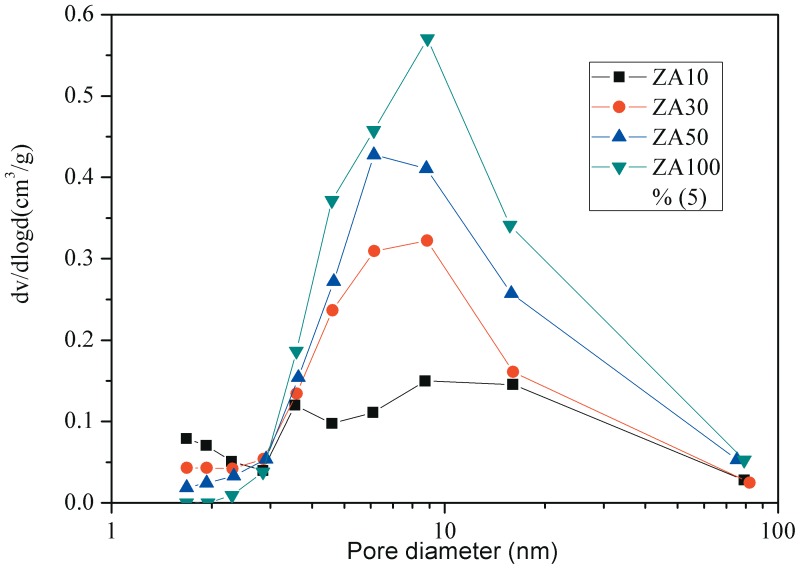
Barrett-Joyner-Halenda (BJH) pore-size distribution curves of different samples. The agglomerated catalysts present a higher mesopore volume with increasing amount of binder.

Nitrogen adsorption-desorption isotherms of different samples are shown in Fig. 1. The Ni/HZSM-5 exhibited a type I isotherm with a plateau at high relative pressure as a result of the microporous nature of the material with limited mesoporosity [9]. With addition of amounts of alumina binder the adsorption-desorption isotherms of these catalysts are similar to that of Ni/HZSM-5. This indicates that pore structure of HZSM-5 is still retained during the process of agglomeration. However, the uptake of N_2_ of these catalysts was gradually increased with the increases in binder content. This was caused by the addition of alumina binder which contains a large number of mesopores created by inter-crystalline voids of alumina crystallites [10]. The pore size distribution profiles of catalysts with different amounts of alumina binder appear in Fig. 2. It is clear that the pore size distributions of ZA10 are similar to that of ZA0. However, with increasing amount of binder, the agglomerated catalyst presents a higher mesopore volume. This mesopore volume is provided by the alumina binder. It has been reported [10] that a well-developed mesopore structure of the catalysts would improve mass and heat transfer in reactions. Table 1 presents the characterization data for different samples. It can be seen that the catalyst surface area and pore diameter tend to decrease with increasing amount of binder.

**Table 1:** Effects of binder content to the structure characteristics of Ni/HZSM-5.

Sample	S_BET_ a (m^2^/g)	V_total_ b (cm^3^/g)	V_microp_ c (cm^3^/g)	V_mesop_ d (cm^3^/g)	Ni loading (wt%)	Ni dispersion (%)
ZA0	284.9	0.1764	0.09819	0.07821	19.6	5.9
ZA 10	274.2	0.2316	0.08886	0.1427	19.7	6.8
ZA30	264.4	0.2855	0.0631	0.2224	19.5	6.5
ZA50	250.3	0.3503	0.04813	0.3022	19.7	5.2
ZA100	237.4	0.404	0.03039	0.3736	19.6	4.5

a Calculated from BET function.

b Evaluated from N_2_ uptake at a relative N_2_ pressure of 0.99.

c T-method micro pore volume.

d V_mesop_ = V_total_−V_microp._

Table 1 also shows that, even with a similar Ni loading, the Ni dispersion on the alumina bound catalysts reduces, demonstrated that the bigger surface area facilitated the dispersion of the Ni species, which is in accordance with the BET results. It should be noticed that ZA0 displayed a poorer mechanical strength than the others. This collapse of ZA0 is probably influencing the homogeneous distribution of Ni species on the catalyst, and thus leading to a poor disperson of Ni than ZA10. It is clear that a larger BET surface area allows for a better metallic dispersion, and a better metallic dispersion is helpful to enhance the catalytic activity of the catalyst.

### XRD

**Figure 3:**
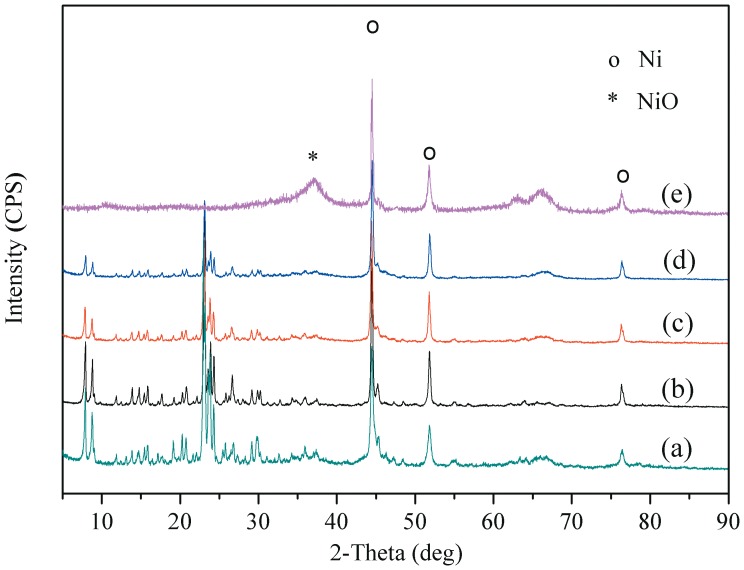
XRD patterns of reduced (a) ZA0, (b) ZA10, (c) ZA30, (d) ZA50, and (e) ZA100. the presences of typical peaks of HZSM-5 (23∼24°) are still maintained for all the agglomerated catalysts. But, the intensities of these diffraction peaks are obviously decreased with increase in the content of the alumina binder.

In order to further investigate the influence of the alumina binder on the catalysts character, the XRD patterns of the reduced catalysts are depicted in Fig. 3. As the alumina binder has nanometer size, the XRD intensity of the alumina is very small and broad compared to that of HZSM-5. Therefore, XRD patterns (not shown) of γ-Al_2_O_3_ are hidden by those of HZSM-5. For the agglomerated catalysts, the presences of typical peaks of HZSM-5 (23∼24°) are still maintained. However, the intensities of these diffraction peaks are obviously decreased with increase in the content of the alumina binder, which may be due to the different scattering properties of the alumina layers [11].

The hydrogenation activities of the metallic catalysts are determined by the number of active sites, and the number of active sites is markedly dependent on the metal particle size [12]. The supported-nickel particle size of various catalysts was calculated by Scherrer equation based on the line of Ni (111) and is summarized in Table 2. It can be found that with the content of alumina binder, the average size of metal active sites is slightly increased. Based on the N_2_ sorption results we think this is probably caused by the decreases in catalyst surface area as discussed before (Table 1).

**Table 2:** The size of the metal sites over catalysts with different content of alumina binder.

Sample	ZA0	ZA10	ZA30	ZA50	ZA100
Size^a^ (nm)	24.6	22.0	23.9	38.0	49.5

a Calculated via Scherrer equation.

### NH_3_-TPD

**Figure 4:**
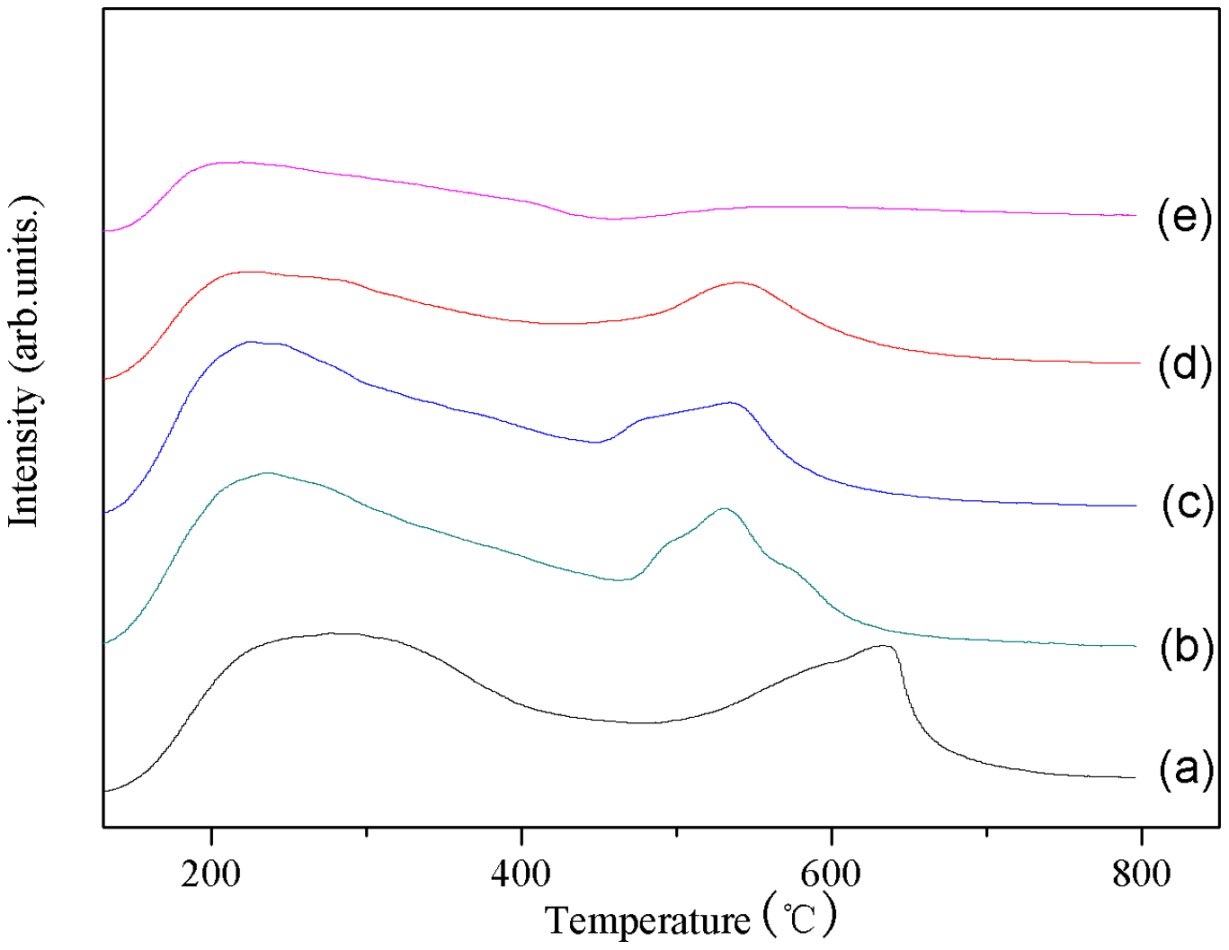
NH_3_-TPD curves for (a) ZA0, (b) ZA10, (c) ZA30, (d) ZA50, and (e) ZA100. All these catalysts exhibited two desorption peaks, i.e., weak and strong acid sites, which give the peaks at around 150–250°C, and 450–550°C respectively.

The hydrodeoxygenation of cyclohexanone is strongly associated with the acidity of catalyst [4], [8]. The temperature-programmed desorption of ammonia (NH_3_-TPD) is used to characterize the influence of alumina content on the acidic properties of catalysts. Fig. 4 shows the NH_3_-TPD profiles for all the catalysts here prepared. Total acidity was defined as the total acid site density, which was obtained by integration of the area under the curve [13]. Values of the corresponding total, weak and strong acid for all the catalysts are given in Table 3. Strong acid was not observed for the ZA100, and with the increase in alumina binder content, the weak acid and strong acid amount of the catalyst gradually decreased. These results revealed that total acid of the alumina binder was clearly lower than that obtained from HZSM-5, and the alumina binder can neutralize the acid sites of catalyst, so as to decrease the acid amount of these agglomerated catalyst. It is worth noting that for the ZA 10 catalyst, an opposite result is observed. As reported in our previous studies [8], during the agglomerated process, some Al species from the binder can migration into the zeolite framework and obviously impact on the acid properties of the catalyst. Thus, we propose that the weak acidity increases of ZA 10 over ZA0 is probably due to the migration of Al species. However, excessive addition of alumina binder will decrease the catalyst acid sites distinctly due to dilution effects.

**Table 3:** NH_3_-TPD results of catalysts with different amounts of alumina binder.

Sample	Weak acid (mmol NH_3_/g)	Strong acid (mmol NH_3_/g)	Total acid (mmol NH_3_/g)
ZA0	0.46	0.19	0.65
ZA 10	0.51	0.12	0.63
ZA30	0.44	0.10	0.54
ZA50	0.33	0.09	0.42
ZA100	0.21	0.05	0.26

### Catalytic activity

**Figure 5:**
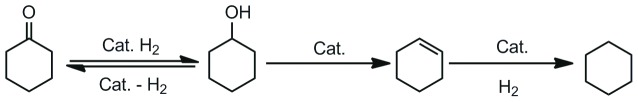
The reaction pathway for HDO of cyclohexanone. Both acid site and metal site are needed for the HDO of cyclohexanone.

It is known that, the Ni/HZSM-5 catalyst is bifunctional and two active centers (the Ni particles and the acid sites) work collaboratively [4], the catalytic process preceded as shown in Fig. 5. The Ni particles can catalyse hydrogenation of the cyclohexanone, and a suitable amount of acidity provided by the support is a potential requirement of the intermediate conversion to olefin. However, the presence of too many acid sites favors the cracking reaction. Moreover, Ni particles with high dispersion are more active those that with lower dispersion.

**Figure 6:**
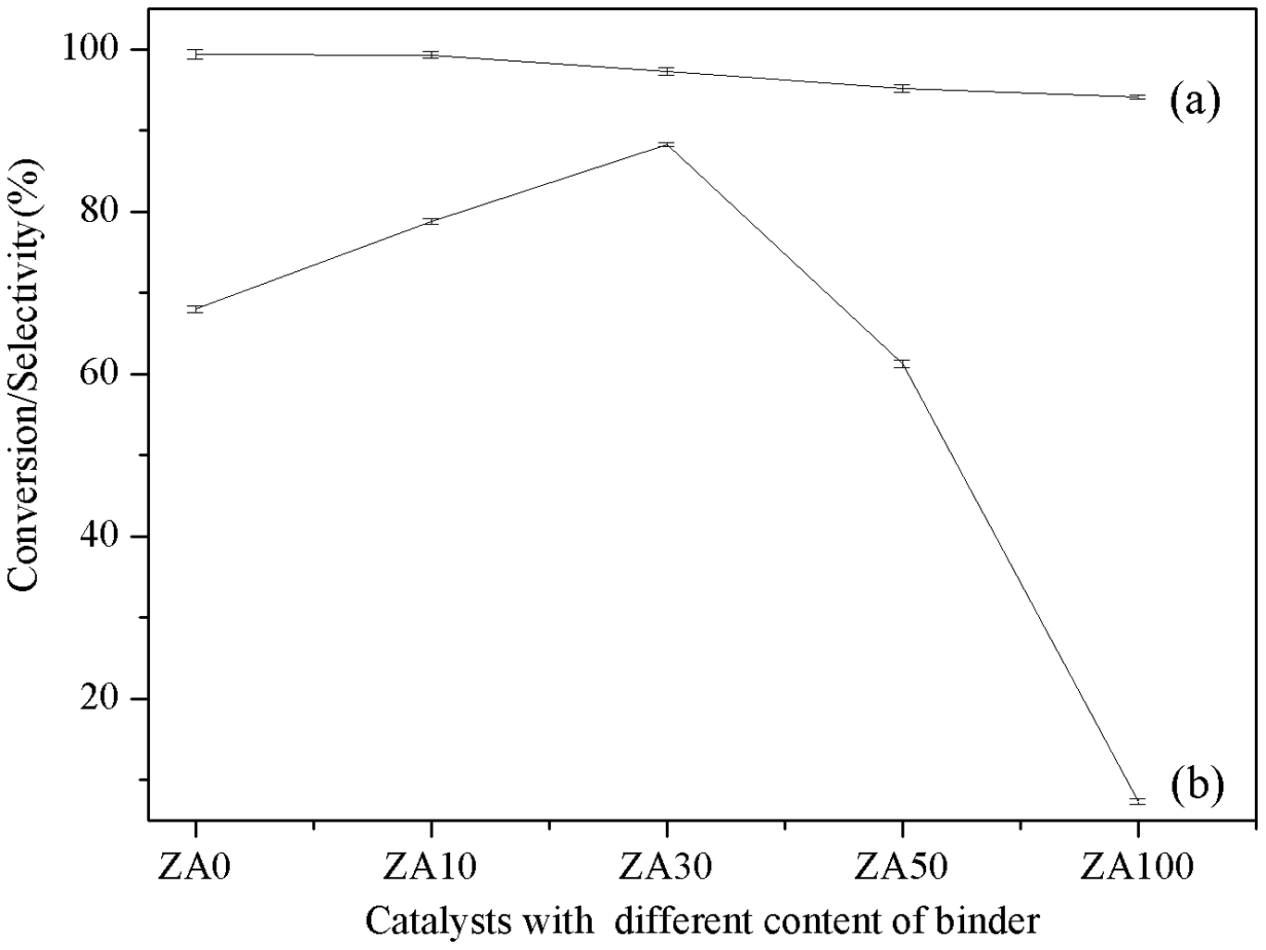
Reaction results of cyclohexanone over different amounts of binder, (a) Conversion, (b) Selcctivity. Fig.6 demonstrated that the reproducibility of the studies is very well.

The catalytic performances of Ni/HZSM-5 with different amounts of alumina binder are shown in Fig. 6 and Table 4. Increasing the alumina binder loading, the cyclohexanone conversion decreases, which can be attributed to changes in the metal dispersion as discussed before. Increasing alumina binder loading, the selectivity towards cyclohexane slightly increased and the maximum value is obtained for the ZA30 catalyst. From Table 3, we know ZA30 has lower acidity than ZA0 and ZA10. Thus, here we propose that the higher activity of ZA30 may be caused by the well-developed mesopore structure of the catalysts, which facilitated the diffusion of cyclohexanol from outside metal sites to inside acid sites, and thus increased the selectivity towards cyclohexane. In addition, this conclusion was in accordance with the TOF data as shown in Table 4, since the TOF data of ZA30 is much bigger than the others. However, the selectivity towards cyclohexane decreases with the continuous increasing content of alumina binder, which may be due to the catalyst’s total acid.

**Table 4:** The influences of binder on catalytic performance of Ni/HZSM-5 for hydrodeoxygenation of cyclohexanone.

Sample	Conversion/%	Selectivity/%					Yield/%	TOF/h^−1^	
		low boilers	Cyclo -hexane	Cyclo -hexene	Cyclo -hexanol	Other	Cyclo -hexane	Ni^0^	NH_3_
ZA0	99.4	8.4	68.0	2.4	18.0	3.2	67.6	3.8	1.2
ZA 10	99.3	6.2	78.8	1.5	10.2	3.3	78.2	3.8	1.4
ZA30	97.3	4.4	88.3	0.6	4.0	2.7	85.9	4.4	1.8
ZA50	95.2	0.0	61.3	0.6	29.0	9.1	58.4	3.7	1.5
ZA100	94.1	0.0	7.3	0.7	83.7	8.3	6.9	0.5	0.3

## Conclusion

The influence of alumina binder content on the catalytic performance of Ni/HZSM-5 for hydrodeoxygenation of cyclohexanone was studied. It can be observed that the amount of surface acid sites and the dispersion properties of Ni particles were decreased as the amount of the alumina binder was increased. Among the catalysts studied, ZA30 exhibits the best catalytic performance, and cyclohexane selectivity of 88.3% was obtained with the corresponding conversion value of 97.3%.
